# Tailored apoptotic vesicles promote bone regeneration by releasing the osteoinductive brake

**DOI:** 10.1038/s41368-024-00293-0

**Published:** 2024-04-16

**Authors:** Yawen Cheng, Yuan Zhu, Yaoshan Liu, Xuenan Liu, Yanan Ding, Deli Li, Xiao Zhang, Yunsong Liu

**Affiliations:** 1 Department of Prosthodontics, Peking University School and Hospital of Stomatology, National Engineering Laboratory for Digital and Material Technology of Stomatology, National Clinical Research Center for Oral Diseases, Beijing Key Laboratory of Digital Stomatology, Beijing, China; 2 Second Clinical Division, Peking University School and Hospital of Stomatology, Beijing, China

**Keywords:** Mesenchymal stem cells, Stem-cell differentiation

## Abstract

Accumulating evidence has demonstrated that apoptotic vesicles (apoVs) derived from mesenchymal stem cells (MSCs; MSC-apoVs) are vital for bone regeneration, and possess superior capabilities compared to MSCs and other extracellular vesicles derived from MSCs (such as exosomes). The osteoinductive effect of MSC-apoVs is attributed to their diverse contents, especially enriched proteins or microRNAs (miRNAs). To optimize their osteoinduction activity, it is necessary to determine the unique cargo profiles of MSC-apoVs. We previously established the protein landscape and identified proteins specific to MSC-apoVs. However, the features and functions of miRNAs enriched in MSC-apoVs are unclear. In this study, we compared MSCs, MSC-apoVs, and MSC-exosomes from two types of MSC. We generated a map of miRNAs specific to MSC-apoVs and identified seven miRNAs specifically enriched in MSC-apoVs compared to MSCs and MSC-exosomes, which we classified as apoV-specific miRNAs. Among these seven specific miRNAs, hsa-miR-4485-3p was the most abundant and stable. Next, we explored its function in apoV-mediated osteoinduction. Unexpectedly, hsa-miR-4485-3p enriched in MSC-apoVs inhibited osteogenesis and promoted adipogenesis by targeting the AKT pathway. Tailored apoVs with downregulated hsa-miR-4485-3p exhibited a greater effect on bone regeneration than control apoVs. Like releasing the brake, we acquired more powerful osteoinductive apoVs. In summary, we identified the miRNA cargos, including miRNAs specific to MSC-apoVs, and generated tailored apoVs with high osteoinduction activity, which is promising in apoV-based therapies for bone regeneration.

## Introduction

Apoptosis is a class of programmed cell death involved in multiple biological processes^1–3^, and in the human body approximately 200 billion cells undergo apoptosis every day^4,5^. During apoptosis, cells secrete a large number of apoptotic vesicles (apoVs)^6^ containing proteins, RNAs, DNAs, and lipids^7,8^. ApoVs play crucial roles in numerous physiological and pathophysiological events^9–11^. As a promising cell source for clinical therapies, mesenchymal stem cells (MSCs) are used to treat several diseases^12–14^. ApoVs derived from MSCs (MSC-apoVs) contribute to the efficacy of MSC-based therapeutics^15–17^. Moreover, the direct application of MSC-apoVs shows therapeutic potential for a variety of diseases^18–25^. We have reported the efficacy of MSC-apoVs for the treatment of bone loss^26^, wound healing^27^, hemophilia A^28^, type 2 diabetes^29^, multiple myeloma^30^, traumatic hemorrhage^31^, and arthritis^32^. Therefore, MSC-apoV therapeutics have considerable clinical utility.

Research has focused on the use of apoVs in bone regeneration^25,26,33^. Compared to MSCs or other extracellular vesicles (EVs) derived from MSCs (such as exosomes; MSC-exosomes), MSC-apoVs have a higher yield, lower cost, and superior osteoinductivity^34^, and thus show promise for bone regeneration. Similar to other EVs, the osteoinductive effect of apoVs is mediated by the bioactive factors they carry, particularly proteins and microRNAs (miRNAs)^35^. Since apoVs have heterogenous cargoes, the identification of cargo profiles specific to MSC-apoVs would provide mechanistic insight and enable the generation of tailored, more-effective apoVs. We previously established the protein profile and identified protein biomarkers specific to MSC-apoVs^28^. However, the features and functions of the miRNAs enriched in MSC-apoVs remain unknown. MSC-apoVs transfer diverse miRNAs to recipient MSCs, promoting the osteogenesis of these cells in vitro and in vivo^16,26^. The miRNAs specific to MSC-apoVs compared to MSCs and MSC-exosomes, and whether all these miRNAs are responsible for their functions in osteoinduction are unclear.

In this study, we compared MSCs, MSC-apoVs, and MSC-exosomes from human bone marrow mesenchymal stem cells (hBMMSCs) and human adipose mesenchymal stem cells (hASCs). Performing RNA sequencing and bioinformatics analyses, we investigated the miRNA cargoes of MSC-apoVs, and identified seven miRNAs specific to MSC-apoVs but not MSCs and MSC-exosomes. Among them, hsa-miR-4485-3p showed the greatest abundance and highest stability. More interestingly, beyond our expectations, hsa-miR-4485-3p enriched in apoVs inhibited osteogenesis and promoted adipogenesis by targeting the AKT pathway. The most specific miRNA is a brake, instead of an accelerator for osteoinductivity of apoVs, which may play a pivotal role in balancing bone metabolism. Furthermore, tailored apoVs with downregulated hsa-miR-4485-3p indeed exhibited a more powerful osteoinductive effect than control apoVs. Through releasing the osteoinductive brake, we acquired one kind of tailored apoVs for bone regeneration. Taken together, our findings reveal the whole miRNA cargoes, identify the miRNAs specific to MSC-apoVs, and generate tailored apoVs with excellent osteoinductivity. Our findings provide a novel insight into the generation of tailored apoVs and promote the development of tailored apoV-based therapeutics for bone regeneration.

## Results

### Characterization of MSC-apoVs

**Fig. 1:**
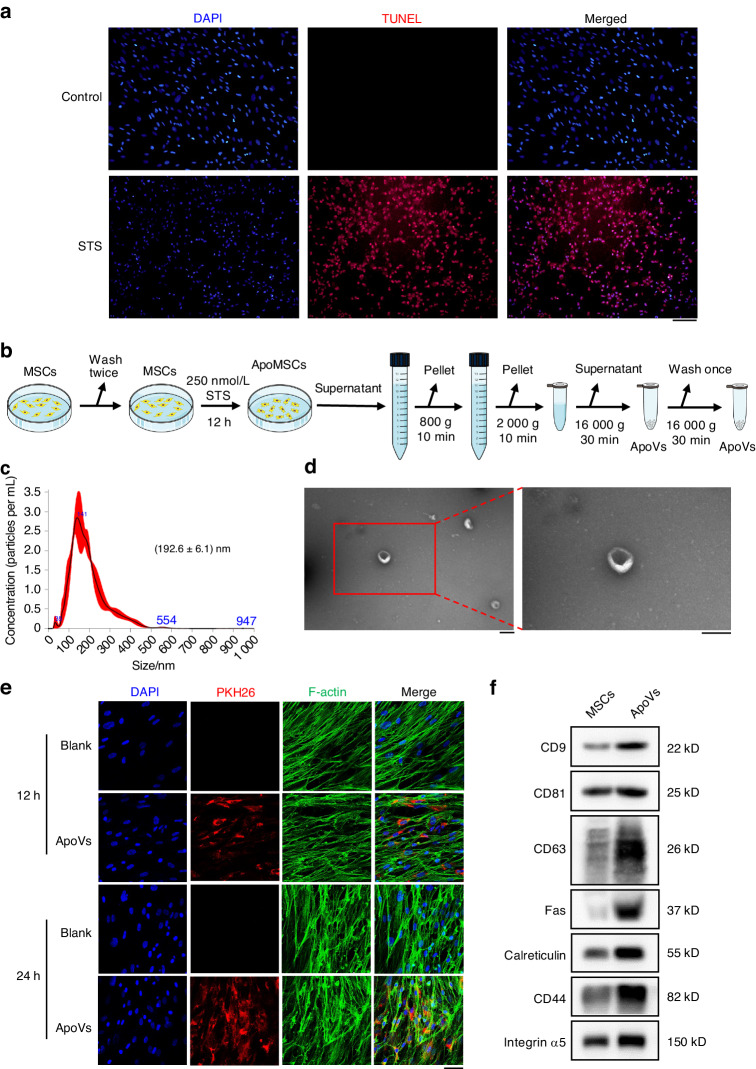
Characterization of MSC-apoVs. **a** TUNEL staining of MSCs induced by STS for 12 h. The nuclei of MSCs were stained with DAPI (blue) while the apoptotic MSCs were TUNEL positive (red). Scale bar, 100 μm. **b** Schematic diagram depicting the gradient centrifugation protocol of MSC-apoV collection. **c** Particle size distribution of MSC-apoVs measured by NTA. The Y-axis represents concentration of MSC-apoVs (particles per mL) and the X-axis represents size of MSC-apoVs (nm). **d** Morphologies of MSC-apoVs under transmission electron microscopic observation. Scale bar, 200 nm. **e** Representative confocal images showing PKH26-labeled MSC-apoVs (red) uptake by MSCs after 12 h and 24 h of co-incubation. The F-actin of MSCs was stained with phalloidin (green) while the nuclei of MSCs were stained with DAPI (blue). Scale bar, 50 μm. **f** Western blot of CD9, CD81, CD63, Fas, Calreticulin, CD44 and Integrin α-5 in MSCs and MSC-apoVs. ApoMSCs, apoptotic MSCs

Staurosporine (STS) was used to induce apoptosis of MSCs. Most MSCs were TUNEL positive after treatment with STS for 12 h (Fig. 1a). Next, we carried out differential centrifugation to extract MSC-apoVs, and the schematic of the isolation protocol was shown in Fig. 1b. Nanoparticle tracking analysis (NTA) showed that the size distribution of MSC-apoVs was 100 to 1 000 nm, and the average diameter was about 192.6 nm. Transmission electron microscopy showed that they were cup-shaped extracellular vesicles of approximately 200 nm in average diameter (Fig. 1c, d). To assess the uptake of MSC-apoVs by MSCs, confocal microscopy was performed after co-incubation of PKH-26-labeled MSC-apoVs and MSCs. F-actin was stained with phalloidin and nuclei with DAPI. MSC-apoVs were internalized by MSCs after 12 h and their abundance in MSCs was increased at 24 h (Fig. 1e). Western blotting (Fig. 1f) revealed that MSC-apoVs contained high levels of CD9, CD81, CD63, Fas, calreticulin, CD44, and integrin α-5, which are markers of apoVs^28^. Therefore, MSC-apoVs had properties consistent with apoVs in terms of morphology, size, biomarkers, and efferocytosis by MSCs^26^.

### Specific miRNA profile of MSC-apoVs compared to MSCs and MSC-exosomes

**Fig. 2:**
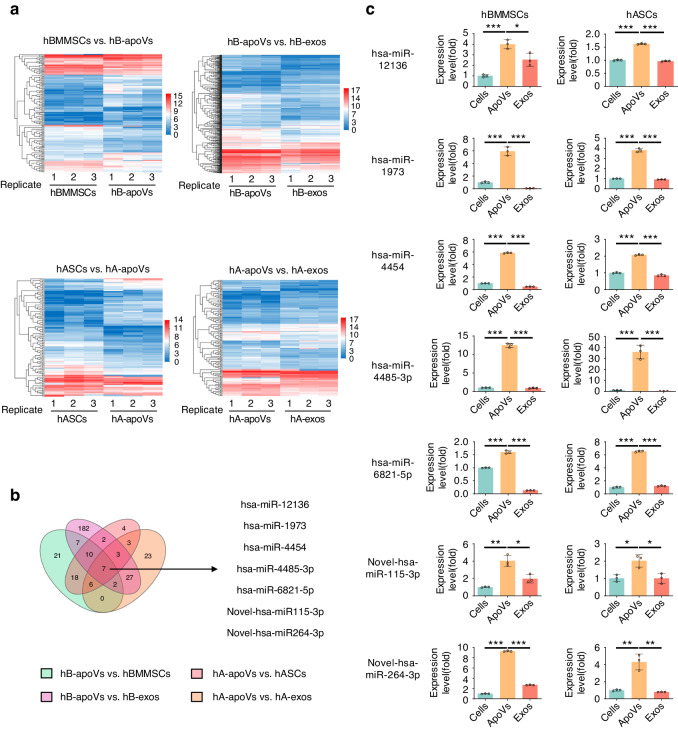
Distinct miRNA profile between MSCs, MSC-apoVs and MSC-exosomes determined by small RNA sequencing analysis. **a** Cluster heatmaps of DEGs among hBMMSCs, hB-apoVs, hB-exos, and hASCs, hA-apoVs, hA-exos. The vertical axis represents miRNAs. Enrichment is depicted in red and depletion is depicted in blue. **b** Venn diagram representing the number of unique and overlapping miRNAs and the novel candidates of unique miRNAs in MSC-apoVs. **c** Relative expression levels of unique miRNA candidates in MSC-apoVs compared to MSCs and MSC-exos. hB-apoVs: hBMMSC-derived apoVs; hB-exos: hBMMSC-derived exosomes; hA-apoVs: hASC-derived apoVs; hA-exos: hASC-derived exosomes. Results are presented as the mean ± standard deviation (*n* = 3). *P < 0.05; **P < 0.01; ***P < 0.001

hBMMSCs and hASCs were subjected to small RNA sequencing. ApoVs derived from hBMMSCs and hASCs were used for miRNA analysis, and parental MSCs and exosomes were used as the controls. In all, 3 280 miRNAs were identified by small RNA sequencing analysis and their expression levels in hBMMSCs, hBMMSC-derived apoVs, hBMMSC-derived exosomes, hASCs, hASC-derived apoVs and hASC-derived exosomes were analyzed. The top 20 highly expressed miRNAs in hBMMSC-derived apoVs and hASC-derived apoVs are listed in Table S1, S2. Up to 85% of the top 20 highly expressed miRNAs overlapped between hBMMSC-derived apoVs and hASC-derived apoVs, suggesting considerable consistency in the miRNA profiles of MSC-apoVs. Adjusted *P*-value < 0.05 and fold-change ≥ 2 were set as the thresholds for differential expression. Cluster heatmaps of differentially expressed genes (DEGs) among hBMMSCs, hBMMSC-derived apoVs, hBMMSC-derived exosomes, and hASCs, hASC-derived apoVs, hASC-derived exosomes were shown in Fig. 2a. 71 miRNAs were upregulated and 66 miRNAs were downregulated in hBMMSC-derived apoVs compared to hBMMSCs; 240 miRNAs were upregulated and 123 miRNAs were downregulated in hBMMSC-derived apoVs compared to hBMMSC-derived exosomes. In the meanwhile, 53 miRNAs were upregulated and 67 miRNAs were downregulated in hASC-derived apoVs compared to hASCs. 71 miRNAs were upregulated and 67 miRNAs were downregulated in hASC-derived apoVs compared to hASC-dervied exosomes (Table S3). The differentially expressed miRNAs detected between pairwise comparisons were further intersected, and seven miRNAs, namely hsa-miR-12136, hsa-miR-1973, hsa-miR-4454, hsa-miR-4485-3p, hsa-miR-6821-5p, novel-hsa-miR-115-3p, and novel-hsa-miR-264-3p, were screened out as a subset of miRNAs which is anticipated to be the candidate of miRNA exhibiting unique expression patterns in MSC-apoVs compared to MSCs and MSC-exosomes (Fig. 2b). Then we shed light on the enrichment analyses of the seven unique miRNAs in MSC-apoVs. Kyoto Encyclopedia of Genes and Genomes (KEGG) pathway analysis (Fig. S1a) showed that “Transport and catabolism” within the “Cellular Processes” domain; “Signal transduction” within the “Environmental Information Processing” domain; “Folding, sorting and degradation” and “Translation” within the “Genetic Information Processing” domain; “Infectious diseases: Viral” and “Cancers: Overview” within the “Human Diseases” domain; “Global and overview maps” within the “Metabolism” domain; and “Immune system”, “Endocrine system” within the “Organismal Systems” domain are predicted to be related to the seven miRNAs. Gene ontology (GO) analysis (Fig. S1b) revealed that “cellular process” in the field of “biological process”, “cell” and “cell part” in the field of “cellular component”, and “binding”, “catalytic activity” in the field of “molecular function” were associated with these seven miRNAs. These results indicated that miRNAs enriched in MSC-apoVs play important roles in a variety of physiological and pathological processes. We then verified the expression levels of the seven candidate miRNAs by qRT-PCR. The results showed that all seven candidates are specifically enriched in MSC-apoVs compared to MSCs and MSC-exosomes (including hBMMSCs and hASCs). Among them, hsa-miR-4485-3p emerged as a lead candidate that showed highest expression in MSC-apoVs compared to MSCs and MSC-exosomes (Fig. 2c). Therefore, we chose hsa-miR-4485-3p for further functional analysis. In summary, MSCs, MSC-apoVs and MSC-exosomes possess distinct miRNA profiles, and seven unique miRNAs that are specifically enriched in MSC-apoVs compared to MSCs and MSC-exosomes were screened out.

### ApoVs overexpressing hsa-miR-4485-3p inhibited osteogenic differentiation and promoted adipogenic differentiation of MSCs in vitro

Next, we investigated the function of hsa-miR-4485-3p in the osteogenic and adipogenic differentiation of MSCs. We performed qRT-PCR to examine the involvement of hsa-miR-4485-3p in these processes. The expression level of hsa-miR-4485-3p was initially high, then decreased and subsequently increased during osteogenic induction, while it was initially low and upregulated afterward during adipogenic induction (Fig. S2a, b). We transfected MSCs with lentiviruses to overexpress hsa-miR-4485-3p (mimics) and RNA oligo to knockdown hsa-miR-4485-3p (inhibitor). qRT-PCR showed that the expression level of hsa-miR-4485-3p in MSC-apoVs was upregulated or downregulated by hsa-miR-4485-3p overexpression or knockdown, respectively, in the parent cells (Fig. S2c–f). To investigate the optimal concentration of MSC-apoVs in vitro, we established a 62.5, 125, 250, 500, and 1 000 ng/mL apoV concentration gradient. A CCK-8 assay revealed that 0-250 ng/mL mi-NC, inhi-NC, mimics, and inhibitor apoVs did not affect the viability of MSCs. At 500 ng/mL and 1 000 ng/mL, apoVs impaired the viability of MSCs (Fig. S2g, h). Analysis of the mRNA levels of the osteogenic differentiation marker *RUNX2* and the adipogenic differentiation marker *PPARγ* revealed that mimics and inhibitor apoV solution at 250 ng/mL had greater effects on the osteogenic and adipogenic activities of MSCs compared to control apoVs after 7 days (Fig. S2i-l). Therefore, the optimal concentration of apoVs was 250 ng/mL; this concentration was used in subsequent in vitro experiments.

**Fig. 3:**
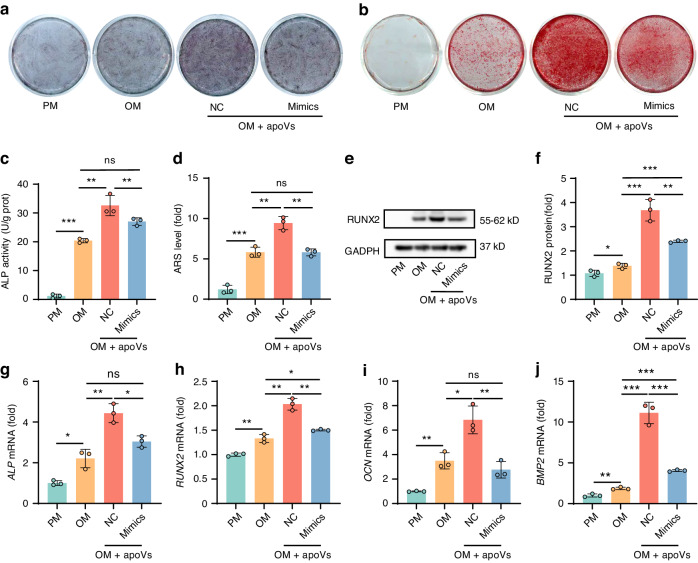
hsa-miR-4485-3p overexpressed apoVs inhibited osteogenic differentiation of MSCs in vitro. **a**–**d** ALP staining (**a**) and ALP activity quantification (**c**) of MSCs after osteogenic induction for 7 days, ARS staining (**b**) and ARS level quantification (**d**) of MSCs after osteogenic induction for 14 days, treated with PM, OM, OM + NC apoVs and OM + mimics apoVs. **e**, **f** Western blot (**e**) and protein expression level quantification (**f**) of RUNX2 in PM, OM, OM + NC apoVs, and OM + mimics apoVs groups after 7 days of treatment. **g**–**j** The relative mRNA expression level of the osteogenic markers *ALP* (**g**) and *RUNX2* (**h**) on day 7, *OCN* (**i**) and *BMP2* (**j**) on day 14 of PM, OM, OM + NC apoVs and OM + mimics apoVs groups determined by qRT-PCR. PM Proliferation medium, OM Osteogenic medium, NC Negative control apoVs; mimics, hsa-miR-4485-3p overexpressed apoVs. Results are presented as the mean ± standard deviation (*n* = 3). ns, *P* > 0.05; *P < 0.05; **P < 0.01; ***P < 0.001

ALP staining (Fig. 3a) and ARS staining (Fig. 3b) of MSCs indicated that the increase of hsa-miR-4485-3p in apoVs inhibited the promotion of osteogenic differentiation. Quantification of ALP activity (Fig. 3c) and ARS level (Fig. 3d) yielded consistent results. Moreover, the RUNX2 protein level declined over 7 days of osteogenesis in the presence of mimics apoVs compared with NC apoVs (Fig. 3e, f). qRT-PCR showed that the *ALP* (Fig. 3g) and *RUNX2* (Fig. 3h) mRNA levels on day 7, and those of *OCN* (Fig. 3i) and *BMP2* (Fig. 3j) on day 14, were low in the presence of mimics apoVs compared with NC apoVs when MSCs were treated with OM.

To further evaluate the effect of hsa-miR-4485-3p in apoVs on adipogenesis, MSCs treated with PM, AM, AM + NC apoVs, and AM+mimics apoVs were subjected to Oil red O staining and quantification (Fig. S3a, b). The upregulation of hsa-miR-4485-3p in apoVs reversed the suppression by apoVs of MSC adipogenesis. As determined by qRT-PCR, the *PPARγ* and *CEBPα* mRNA levels were upregulated in the presence of mimics apoVs (Fig. S3c, d). Furthermore, western blotting of the protein level of PPARγ yielded consistent results (Fig. S3e, f). Therefore, hsa-miR-4485-3p overexpression in apoVs promoted adipogenic differentiation and inhibited osteogenic differentiation of MSCs in vitro.

### ApoVs knockdown hsa-miR-4485-3p enhanced osteogenic differentiation and suppressed adipogenic differentiation of MSCs in vitro

**Fig. 4:**
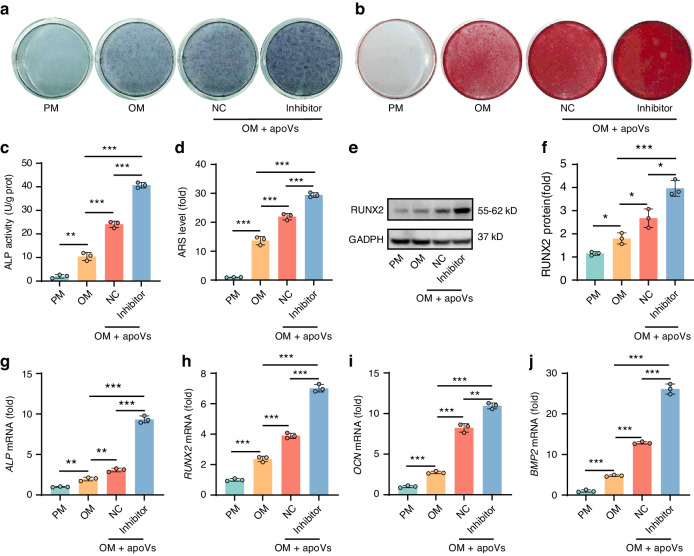
hsa-miR-4485-3p knockdown apoVs enhanced osteogenic differentiation of MSCs in vitro. **a**–**d** ALP staining (**a**) and ALP activity quantification (**c**) of MSCs after osteogenic induction for 7 days, ARS staining (**b**) and ARS level quantification (**d**) of MSCs after osteogenic induction for 14 days, treated with PM, OM, OM + NC apoVs and OM + inhibitor apoVs. **e**, **f** Western blot (**e**) and protein expression level quantification (**f**) of RUNX2 in PM, OM, OM + NC apoVs, and OM + inhibitor apoVs groups after 7 days of treatment. **g**–**j** The relative mRNA expression levels of the osteogenic markers *ALP* (**g**) and *RUNX2* (**h**) on day 7, *OCN* (**i**) and *BMP2* (**j**) on day 14 of PM, OM, OM + NC apoVs and OM + inhibitor apoVs groups determined by qRT-PCR. PM, proliferation medium; OM, osteogenic medium; NC, negative control apoVs; inhibitor, hsa-miR-4485-3p knockdown apoVs. Results are presented as the mean ± standard deviation (*n* = 3). ns, *P* > 0.05; *P < 0.05; **P < 0.01; ***P < 0.001

ALP and ARS staining (Fig. 4a, b) and quantification (Fig. 4c, d), western blotting (Fig. 4e, f) and qRT-PCR (Fig. 4g–j) showed that hsa-miR-4485-3p downregulation in apoVs enhanced the promotion of the osteogenic activity of MSCs. In addition, the loss of hsa-miR-4485-3p in apoVs further inhibited MSC adipogenic differentiation, as shown by Oil red O staining and quantification (Fig. S3g, h), qRT-PCR (Fig. S3i, j) and western blotting (Fig. S3k, l). Therefore, hsa-miR-4485-3p in apoVs inhibited osteogenesis and promoted adipogenesis by MSCs in vitro.

### hsa-miR-4485-3p in apoVs attenuated osteogenic differentiation and enhanced adipogenic differentiation of MSCs in vivo

**Fig. 5:**
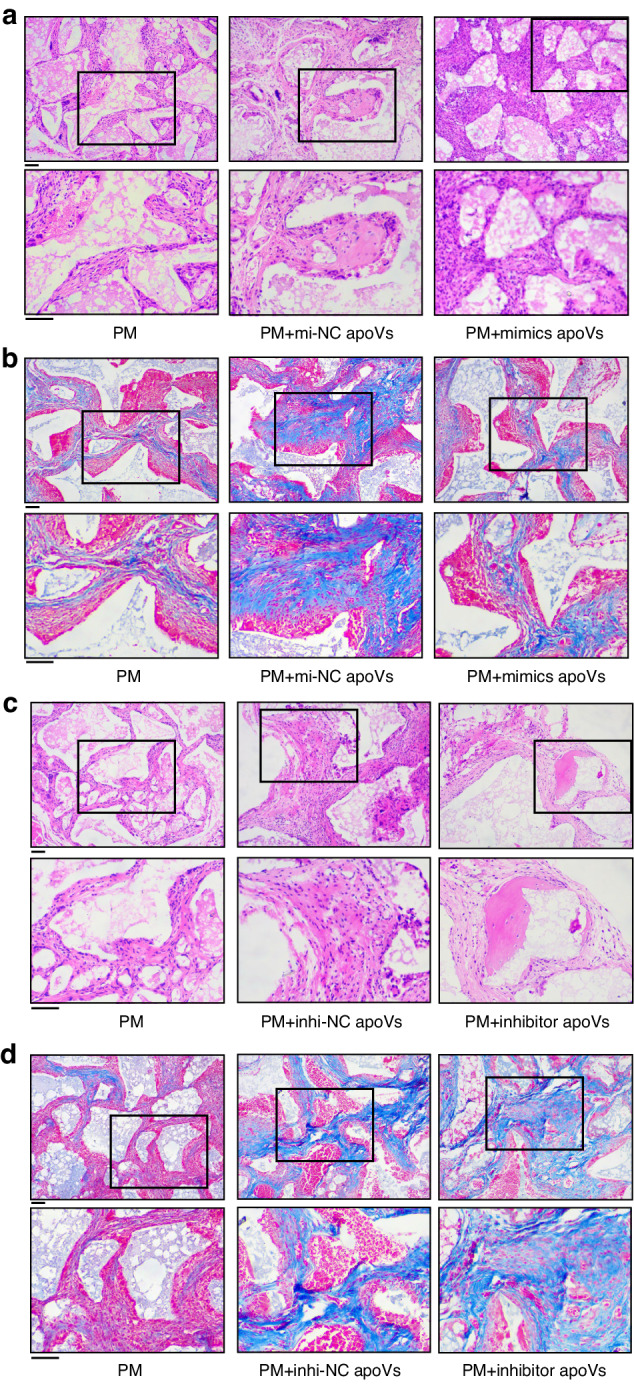
Histological sections of neo-generated bone tissue induced by MSCs treated by hsa-miR-4485-3p overexpressed and knockdown apoVs. Representative microscopic view of H&E staining (**a**) and Masson trichrome staining (**b**) of PM, PM+mi-NC apoVs, and PM+ mimics apoVs groups; H&E staining (**c**) and Masson trichrome staining (**d**) of PM, PM+inhi-NC apoVs and PM+inhibitor apoVs groups. The lower panels show the magnified images of the area indicated by the black lines. Scale bar, 100 μm. PM, proliferation medium; mimics apoVs, hsa-miR-4485-3p overexpressed apoVs; mi-NC apoVs, negative control of mimics apoVs; inhibitor apoVs, hsa-miR-4485-3p knockdown apoVs; inhi-NC apoVs, negative control of inhibitor apoVs

To evaluate the function of hsa-miR-4485-3p in apoVs in vivo, nude mice were implanted with β-TCP carrier scaffold loaded with MSCs cultured in PM, PM + 250 ng/mL inhi-NC apoVs, PM + 250 ng/mL inhibitor apoVs, PM + 250 ng/mL mi-NC apoVs, and PM + 250 ng/mL mimics apoVs in the dorsal subcutaneous area. H&E staining of ectopic bone formation tissues showed that neo-generated bone was significantly reduced (Fig. 5a), and Masson’s trichrome staining (Fig. 5b) revealed that less collagen tissue was formed in the PM+mimics apoVs group compared to the PM+mi-NC apoVs group. In addition, the areas of newly formed bone-like tissue and collagen were increased in the PM+inhibitor apoVs group compared to the PM+inhi-NC group (Fig. 5c, d). Therefore, hsa-miR-4485-3p in apoVs attenuated the osteogenic differentiation of MSCs in vivo.

To further investigate the effect on adipogenesis of hsa-miR-4485-3p in apoVs in vivo, we cultured MSCs in PM, AM, AM + 250 ng/mL inhibitor apoVs, AM + 250 ng/mL inhi-NC apoVs, AM + 250 ng/mL mimics apoVs, and AM + 250 ng/mL mi-NC apoVs separately for 7 days; then we combined them with collagen sponge scaffolds and implanted the scaffolds into nude mice. Staining with H&E and Oil Red O showed that mimics apoVs increased the number of lipid droplets compared to mi-NC apoVs (Fig. S4a, b). Less adipose-like tissue was presented in H&E- and Oil Red O-stained histological sections from the AM+inhibitor apoVs group than AM+inhi-NC apoVs group (Fig. S4c, d). These results suggest that hsa-miR-4485-3p in apoVs enhanced the adipogenic differentiation of MSCs in vivo. In conclusion, hsa-miR-4485-3p in apoVs acts as a switch in MSC fate commitment towards adipogenesis and against osteogenesis in vitro and in vivo.

### hsa-miR-4485-3p in apoVs control MSC fate commitment by regulating the AKT pathway

**Fig. 6:**
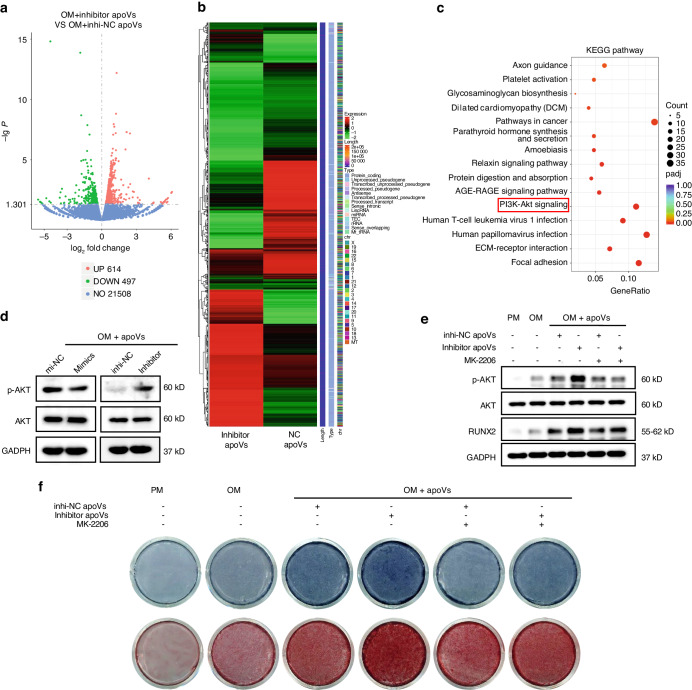
hsa-miR-4485-3p control MSC osteogenesis through regulating AKT pathway. **a** Volcano plot of DEGs between OM+inhibitor apoVs and OM+inhi-NC apoVs groups. *P*-value < 0.05 in combination with fold changeå 1 was set to identify the differential expression. The red dots represent upregulated genes, and the green dots represent downregulated genes in OM + inhibitor apoVs group compared to OM + inhibitor apoVs group. The blue dots represent genes with no significant difference between the two groups. **b** Heatmap of DEGs between OM+inhibitor apoVs and OM+inhi-NC apoVs groups. The vertical axis represents genes. Enrichment is depicted in red and depletion is depicted in green. **c** Kyoto Encyclopedia of Genes and Genomes (KEGG) pathway analysis showed that AKT pathway is up-regulated in the OM + inhibitor apoVs group compared to OM + inhi-NC apoVs group. The Y-axis represents KEGG terms and the X-axis represents enrichment ratio. The color of the bubble represents the enrichment significance and the size of the bubble represents the number of genes. **d** Western blot showed that hsa-miR-4485-3p in apoVs inhibited the AKT signaling pathway in osteogenesis. **e**, **f** Western blot (**e**) and ALP, ARS staining (**f**) results demonstrated that MK-2206 suppressed the AKT signaling pathway and rescue the effect of hsa-miR-4485-3p in osteogenesis. PM proliferation medium; OM osteogenic medium; mimics apoVs, hsa-miR-4485-3p overexpressed apoVs; mi-NC apoVs, negative control of mimics apoVs; inhibitor apoVs, hsa-miR-4485-3p knockdown apoVs; inhi-NC apoVs, negative control of inhibitor apoVs

To provide insight into the mechanism by which hsa-miR-4485-3p in apoVs regulate MSC fate commitment, we carried out RNA sequencing; the DEGs between OM+inhibitor apoVs and OM+inhi-NC apoVs groups were shown as a volcano plot and heatmap in Fig. 6a, b. *P*-value < 0.05 and fold change å 1 were set as the thresholds for differential expression; 614 genes were upregulated and 497 genes were downregulated in OM+inhibitor apoVs group compared to OM+inhi-NC apoVs group. KEGG enrichment analysis of DEGs indicated upregulation of the AKT pathway in the OM+inhibitor apoVs group compared to the OM+inhi-NC apoVs group (Fig. 6c). Next, we conducted western blotting of components of the NF-κB, Wnt, Smad and Erk signaling pathways (Fig. S5), which are related to MSC fate commitment. The AKT signaling pathway was more significantly influenced than the other pathways by hsa-miR-4485-3p in apoVs (Fig. 6d, Fig S6a). Western blotting, ALP and ARS staining results (Fig. 6e, f) showed that MK-2206 reversed the promotion of osteogenesis by inhibitor apoVs. Furthermore, western blotting and ORS staining (Fig. S6b, c) demonstrated that MK-2206 (an inhibitor of the AKT pathway) rescued the suppression of adipogenesis by inhibitor apoVs. In summary, hsa-miR-4485-3p in apoVs suppressed the osteogenic differentiation and promoted adipogenic differentiation of MSCs by regulating the AKT signaling pathway.

### Tailored apoVs are more effective in bone restoration of osteoporosis

MSC-derived hsa-miR-4485-3p knockdown apoVs promoted MSC osteogenic differentiation to a greater extent in vitro and in vivo compared to NC apoVs; hereafter, the former are termed “tailored apoVs” for bone regeneration. We injected DiR-labeled apoVs into the tail vein of mice to monitor the biodistribution of apoVs. Whole-body fluorescence imaging (Fig. S7) revealed that NC apoVs and inhibitor apoVs underwent hepatic metabolism, whereas NC apoVs and inhibitor apoVs were enriched in the femur; the level of enrichment increased for 48 h. Next, we assessed the effect of tailored apoVs on the prevention of osteoporosis in estrogen deficiency mice and on the treatment of osteoporosis in aged mice. We established estrogen deficiency-induced osteoporosis in mice by ovariectomy (OVX) and purchased mice with senescence-induced osteoporosis. We injected phosphate-buffered saline (PBS), NC apoVs, and inhibitor apoVs into the tail vein at 20 μg per 30 g body weight, a dose confirmed by our previous study to be biocompatible and effective^26^. In detail, we set concentration gradients, namely 10, 20, 40 μg per 30 g weight. The micro-CT and BMD parameters of femurs of osteoporosis mice showed that 20 μg apoVs per 30 g weight was the most effective concentration in bone repair^26^.

**Fig. 7:**
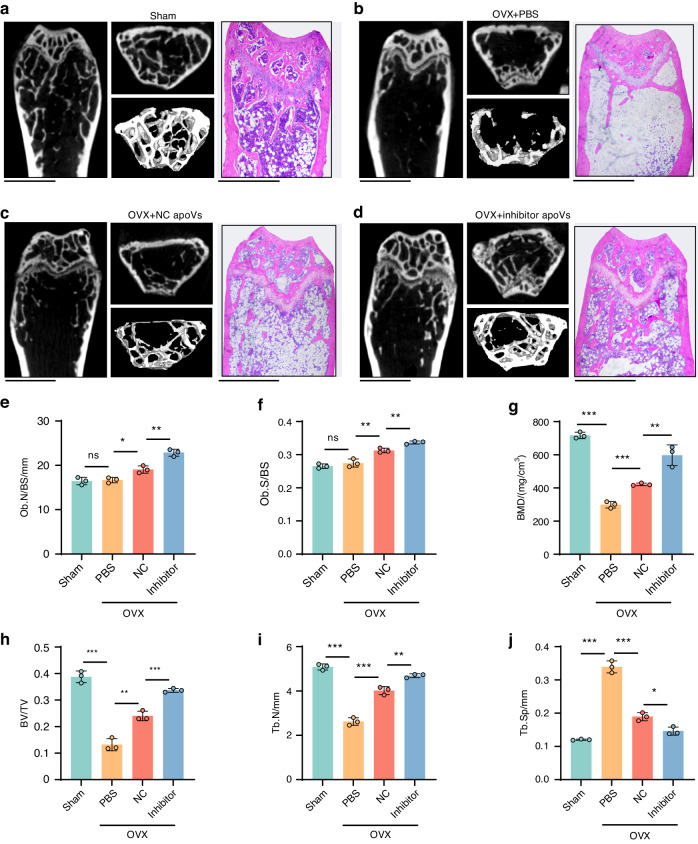
Tailored apoVs are more effective than normal apoVs in bone restoration of estrogen deficiency induced-osteoporosis. **a**–**d** Representative micro-CT images of longitudinal section, cross section, 3D reconstruction view and representative H&E staining of femurs in sham (**a**), OVX (**b**), OVX + NC apoVs (**c**) and OVX + inhibitor apoVs (**d**) groups. Scale bar, 1.0 mm. Histomorphometric examination of Ob.N/BS (e), Ob.S/BS (**f**) parameters from histological sections, BMD (**e**), BV/TV (**f**), Tb. N (**g**) and Tb. Sp (**h**) parameters from micro-CT, of femurs taken from sham mice, and OVX mice treated with PBS, NC apoVs and inhibitor apoVs. Sham sham operation; OVX, ovariectomy operation; PBS, injection with PBS solution; NC, injection with negative control apoV solution; inhibitor, injection with hsa-miR-4485-3p knockdown apoV solution. Results are presented as the mean ± standard deviation (*n* = 3 per group). ns, *P* > 0.05; *P < 0.05; **P < 0.01; ***P < 0.001

**Fig. 8:**
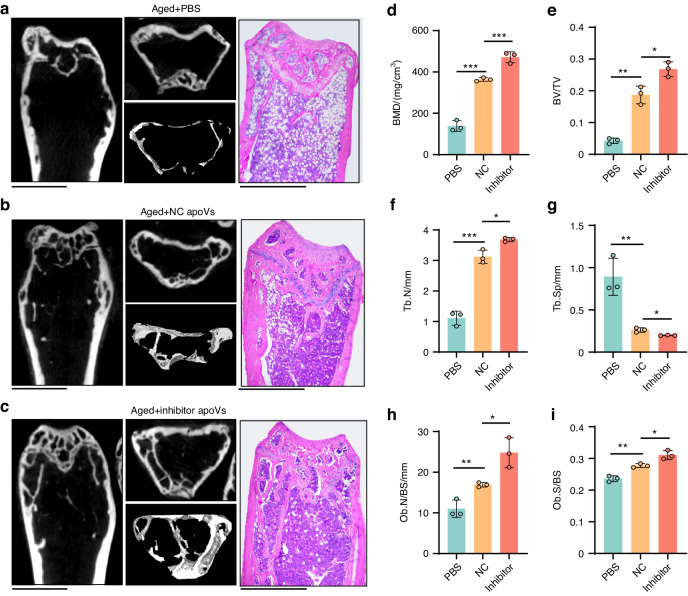
Tailored apoVs are more effective than normal apoVs in bone restoration of osteoporosis induced by aging. **a**–**c** Representative micro-CT images of longitudinal section, cross section and 3D reconstruction view and representative H&E staining of femurs in PBS(**a**), NC (**b**) and inhibitor (**c**) groups. Scale bar, 1.0 mm. Histomorphometric examination of BMD (**d**), BV/TV (**e**), Tb. N (**f**) and Tb. Sp (**g**), Ob. N/BS (**h**), Ob. S/BS (**i**) parameters of femurs taken from aging mice treated with PBS, NC apoVs and inhibitor apoVs. PBS, injection with PBS solution; NC, injection with negative control apoV solution; inhibitor, injection with hsa-miR-4485-3p knockdown apoV solution. Results are presented as the mean ± standard deviation (*n* = 3 per group). ns, *P* > 0.05; *P < 0.05; **P < 0.01; ***P < 0.001

Eight weeks later, the femurs were collected and scanned by micro-CT. The longitudinal section, cross-section, and three-dimensional reconstruction views demonstrated massive bone deterioration in the femurs of OVX and aged mice, and trabecular bone restoration was greater in the inhibitor apoVs group compared to the NC apoVs group in both models. H&E staining exhibited that the restoration of bone impairment in both models was more obvious in the inhibitor apoVs group than the NC apoVs group (Fig. 7a–d, Fig. 8a–c). The parameters of osteoblast number/bone surface (Ob.N/BS) and osteoblast surface/bone surface (Ob.S/BS) showed that osteoblast differentiation increased in mice treated with tailored apoVs compared to mice in control groups (Fig. 7e, f, Fig. 8h, i). In the histomorphometric analysis of micro-CT, the bone mineral density (BMD), bone volume/total volume (BV/TV), and trabecular number (Tb. N) were higher, and trabecular spacing (Tb. Sp) was lower, in experimental groups (Fig. 7g–j, Fig. 8d–g). Therefore, tailored apoVs can be delivered to long bones to ameliorate the loss of bone mass in osteoporosis more effectively.

### PLGA/pDA-tailored apoVs scaffolds promoted more bone formation in calvarial defects of rats

**Fig. 9:**
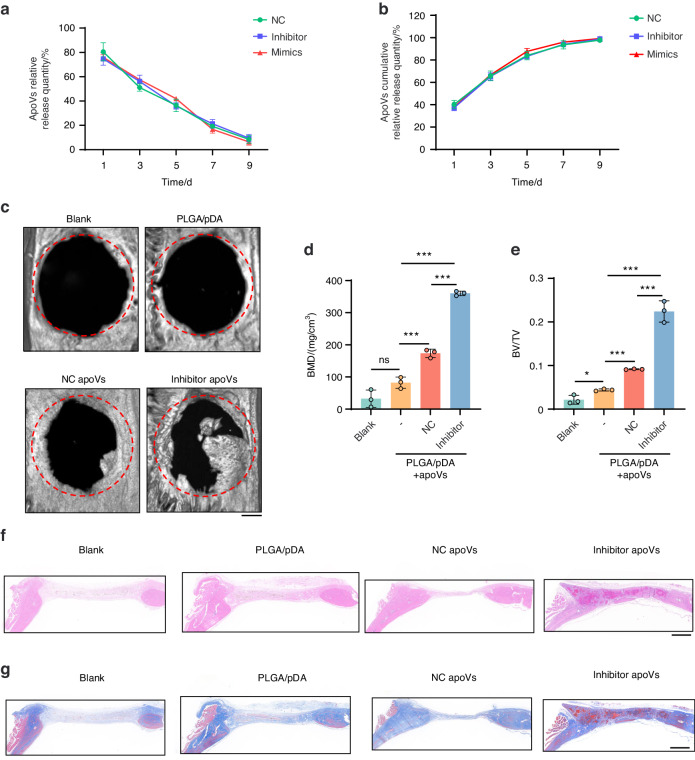
PLGA/pDA-tailored apoVs scaffold promoted bone formation in rat calvarial defects. **a** ApoV relative release quantity of PLGA/pDA-apoVs at day 1, 3, 5, 7, 9. **b** ApoV cumulative relative release quantity of PLGA/pDA-apoVs at day 1, 3, 5, 7, 9. **c** Representative micro-CT images of calvarial defects in blank, PLGA/pDA, PLGA/pDA-NC apoVs and PLGA/pDA-inhibitor apoVs groups 8 weeks after operation. Scale bar, 1 mm. **d**, **e** Histomorphometric examination of BMD (**d**), BV/TV (**e**) parameters of calvarial defects. **f**, **g** Representative H&E (**f**) and Masson (**g**) staining of blank, PLGA/pDA, PLGA/pDA-NC apoVs and PLGA/pDA-inhibitor apoVs groups. Scale bar, 1 mm. inhibitor apoVs, hsa-miR-4485-3p knockdown apoVs; NC apoVs, negative control apoVs. Results are presented as the mean ± standard deviation (*n* = 3 per group). ns, *P* > 0.05; *P < 0.05; ***P < 0.001

ApoVs become integrated in PLGA/pDA-apoV composite scaffolds, enabling promotion of bone formation in rat calvarial defects^26^. In this study, PLGA/pDA-apoV composite scaffolds slowly released apoVs (Fig. 9a, b). We established a rat skull-defect model to assess the function of tailored apoVs in localized bone loss. In micro-CT images (Fig. 9c), there were no signs of healing in the blank group. Rats implanted with the PLGA/pDA scaffolds showed formation of new bone tissue at the edges of the skull defects. Those implanted with PLGA/pDA-NC apoV scaffolds showed formation of more new bone tissue, and rats with the PLGA/pDA-tailored apoV scaffolds had a significantly greater amount of new bone. The BMD and BV/TV values (Fig. 9d, e) indicated more new bone tissue generation in the rats with PLGA/pDA-tailored apoV scaffolds than in those with PLGA/pDA-NC apoV scaffolds. H&E and Masson’s trichrome staining (Fig. 9f, g) confirmed the superior promotion of new bone formation in the calvarial defects of rats by the PLGA/pDA-tailored apoV scaffolds.

## Discussion

Apoptosis is an indispensable physiological activity that maintains homeostasis and plays a pivotal role in tissue regeneration. MSC is one of the most appealing cell sources used in bone tissue engineering, and recent advances in exploring the functional products of MSC apoptosis attracted widespread attention to MSC-apoVs. The various advantages of MSC-apoVs exhibit the potential to break through the limitations in the large-scale clinical translation of cell- and exosome-based therapeutics^36,37^.

The heterogeneity in bioactive constituents of MSC-apoVs results in them having a variety of functions^35^ and may impede their use for the precisive treatment of bone-related diseases. Among the diverse contents of MSC-apoVs, miRNAs are important; indeed, the expression of 60% of mammalian genes is regulated by miRNAs^38^. Several miRNAs are regulators of MSC osteogenesis^39,40^. Therefore, miRNAs specific to MSC-apoVs were identified and characterized. We performed small RNA sequencing of MSCs (including hBMMSCs and hASCs), MSC-apoVs and MSC-exosomes comprehensively. In all, 3280 miRNAs were identified and their expression levels were analyzed. The differentially expressed miRNAs were intersected and seven miRNAs specifically enriched in MSC-apoVs compared to MSCs and MSC-exosomes were screened out and verified through qRT-PCR. hBMMSCs and hASCs had similar miRNA profiles. However, the miRNA expression levels differed among MSCs, MSC-apoVs and MSC-exosomes. The number of distinct miRNAs is typically between 100-400. We previously identified more than 5 600 proteins, among which several thousand were specific to MSC-apoVs compared to MSC-exosomes^28^. Therefore, the distinction is more obvious in proteomics than in miRNA profiles between MSC-apoVs and MSC-exosomes, suggesting that miRNAs and proteins have different mechanisms of sorting into apoVs^41^. The secretion of miRNAs into exosomes depends on EXOmotifs, the insertion of which leads to a reduction in the expression levels of target genes in distant cells^42^. Zheng et al. identified proteins with conserved EV-sorting abilities and showed that the genetic fusion of specific proteins to scaffold proteins enables the generation of stable EV-delivery vehicles^43^. The above studies suggest that the efficacy of EVs could be improved by tinkering with their cargo-loading mechanisms. ApoV is a next-generation drug-delivery modality, so it is vital to analyze the mechanisms underlying the loading of specific cargoes into apoVs to improve apoV-based therapeutics.

MSC-apoVs regulate bone metabolism and promote bone repair as a whole^16,26,44^. Hsa-miR-4485-3p, the most abundant and stable of the seven apoV-specific miRNAs, was expected to be an “accelerator” for bone restoration in our preliminary assumption. Hsa-miR-4485-3p is 20 nucleotides long and is produced from the hsa-mir-4485 precursor. Hsa-miR-4485 is related to cancer^45^, infectious diseases^46,47^, and bone-fracture healing^48^. Very interestingly and unexpectedly, we found hsa-miR-4485-3p in MSC-apoVs actually act as an inhibitor of osteogenesis and an enhancer of adipogenesis, that is, a “brake” for osteoindictivity of MSC-apoVs. Our results showed that in the early stages of MSC osteogenesis, the expression level of hsa-miR-4485-3p decreased; however, it remarkably increased in the late stages. Therefore, the underlying reason could be that hsa-miR-4485-3p in apoVs exerts a substantial effect on balancing bone metabolism.

**Fig. 10:**
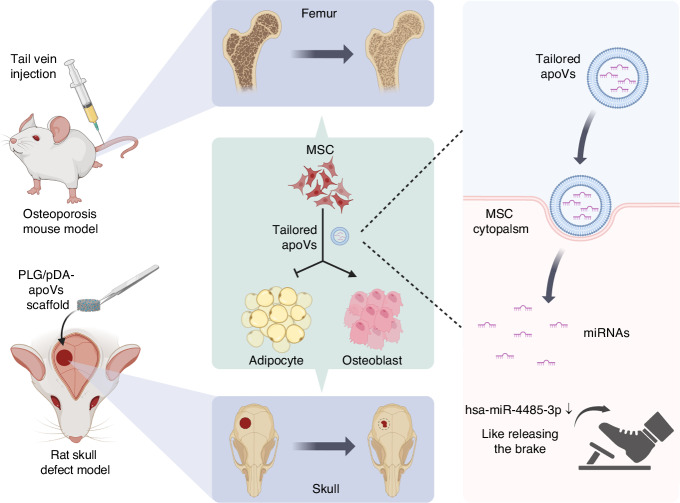
Mechanistic diagrams of tailored apoVs in treating osteoporosis and skull defects by reducing hsa-miR-4485-3p, which control MSC fate commitment towards adipogenesis and against osteogenesis, like releasing the brake

As key mediators of intercellular communication, apoVs carry, protect, and transport specific miRNAs to modulate bone homeostasis at distant sites^49^. Because hsa-miR-4485-3p knockdown MSC-apoVs showed an enhanced osteoinductive effect, so it is exploited as tailored apoVs in bone regeneration. Systemic infusion of tailored apoVs had a more effective therapeutic potential for osteoporosis caused by estrogen deficiency and senescence by regulating the fate decision of MSCs^50^. Moreover, tailored apoVs combined with a PLGA/pDA scaffold significantly enhanced new bone formation in maxillofacial bone defects. In the present medical context, the importance of precision medicine has gained more and more attention. Notably, most studies on tailored extracellular vesicles have focused on the addition of promoters of osteogenesis^51,52^, like stepping on the accelerator of osteogenesis. However, our findings indicated that the subtraction of a particular component, hsa-miR-4485-3p, had an additive effect on the osteoinductive ability of MSC-apoVs, like releasing the brake (Fig. 10). This approach presents new avenues to improve the therapeutic efficiency of apoVs for use in precision medicine.

This study had several limitations. First, the direct target gene of hsa-miR-4485-3p in MSC-apoVs and the function of other apoV-specific miRNAs remain elusive and will be the subject of further research. Second, we confirmed that hsa-miR-4485-3p overexpressing MSC-apoVs promoted MSC adipogenetic differentiation in vivo. Considering that cutaneous damage is also a thorny issue in regenerative medicine^53,54^, problems need to be addressed whether hsa-miR-4485-3p overexpressed apoVs are more effective in soft tissue engineering. Thirdly, in addition to the osteo-adipogenic differentiation imbalance observed in MSCs, the senescence of MSCs is also associated with osteoporosis caused by aging^55,56^. The potential of tailored apoVs to reverse MSC senescence remains uncertain. We aim to address this hypothesis through subsequent research using both in vivo and in vitro experimental approaches. Besides the above limitations, the distribution and pharmacokinetics of tailored apoVs need to be further discussed in our subsequent research if they are to be used clinically. What’s more, this study offers a novel perspective, suggesting that the production of more potent apoVs for osteoinduction might be feasible by adding the accelerator and releasing the brake simultaneously. This “working along both lines” approach merits subsequent investigation and has promising clinical prospects.

In conclusion, our investigation expanded our understanding of MSC-apoV specific miRNA profile and paved the way for novel strategies to achieve precision treatment in regenerative medicine.

## Materials and methods

### Proliferation, osteogenic and adipogenic induction of human MSCs

hBMMSCs and hASCs were purchased from Science Cell Research Laboratories (San Diego, CA). hBMMSCs were cultured in proliferation medium (PM) containing 10% FBS, α-MEM, and 1% streptomycin-penicillin. hASCs were cultured in PM containing 10% FBS, DMEM, and 1% streptomycin-penicillin. Osteogenic medium (OM) was standard PM with 0.2 mmol/L ascorbic acid, 10 mmol/L β-glycerophosphate, and 10 nmol/L dexamethasone. Adipogenic medium (AM) was standard PM with 200 μmol/L indomethacin, 100 nM dexamethasone, 50 nmol/L insulin and 500 μmol/L 3-isobutyl-1-methylxanthin.

### Induction of apoptosis, isolation, and identification of apoVs

Culture medium was substituted for α-MEM/DMEM with 500 nmol/L STS (Enzo Life Sciences) to induce apoptosis of MSCs. MSCs were labeled using the TUNEL Apoptosis Detection Kit (Applygen, C000320) and rhodamine fluorescein (red) dUTP, and apoptosis was observed using a fluorescence microscopy (Olympus). After 12 h, apoVs were extracted by differential centrifugation (Fig. 1b)^39^. The Pierce BCA Assay Kit (Thermo Scientific) was used to quantify the apoVs concentration.

ApoVs were deposited onto a carbon-coated copper net, stained twice with 1% uranyl acetate, and their morphology was visualized using an HT7700 transmission electron microscopy (Hitachi). The size distributions were evaluated using the Nano Sight NS300 (Malvern) according to the manufacturer’s instructions. ApoV markers (CD9, CD81, CD63, Fas, calreticulin, CD44, and integrin α-5) were detected by Western blotting.

### Assay of apoV uptake in vitro

ApoVs were labeled with PKH-26 using the Red Fluorescent Cell Labeling Kit (Umibio). Next, they were washed and the supernatant was collected as the negative control (NC). Labeled apoVs and NC (250 ng/mL) were incubated with MSCs for 12 or 24 h, and 5 μg/mL phalloidin (Sigma-Aldrich) and 6-diamidine-2-phenylindole (DAPI) were added. Fluorescence imaging was performed using a confocal LSM 5 EXCITER microscope (Carl Zeiss).

### Isolation of exosomes

Exosomes were removed from fetal bovine serum (FBS) by ultracentrifugation at 120 000 g for 18 h. Cells were cultured in exosome-free medium for 2 days, and the supernatant was centrifuged at 300 g for 10 min, 3 000 g for 10 min, and 20 000 g for 30 min. Leftover supernatant was passed through a 0.22 μm filter (Millipore) and ultracentrifuged at 120 000 g for 120 min to isolate exosomes.

### Lentivirus infection

Recombined lentivirus overexpressing hsa-miR-4485-3p (mimics) and the NC (mi-NC) were purchased from GenePhama (Suzhou, China). The sequences were: mimics: 5’-TAACGGCCGCGGTACCCTAA-3’; mi-NC: 5’-TTCTCCGAACGTGTCACGT-3’. For transfection, cells at 30%–40% confluence were exposed to the viral supernatant. After 2 days, 1 mg/mL puromycin (Sigma-Aldrich) was added to select stably transfected cells.

### Transient infection with miRNAs and plasmids

An RNA oligo with a 2’-ome modification to knockdown hsa-miR-4485-3p (inhibitor) and its NC (inhi-NC) were obtained from GenePhama (Suzhou, China). The sequences were: inhibitor: 5’-UUAGGGUACCGCGGCCGUUA-3’; inhi-NC: 5’-CAGUACUUUUGUGUAGUACAA-3’. The cells were transfected using Lipofectamine 3000 (Invitrogen).

### Cell counting kit-8 assay

MSCs were cultured in PM or PM supplemented with 62.5, 125, 250, and 500 ng/mL MSC-apoVs. MSC viability of MSCs was evaluated with the Cell Counting Kit-8 (CCK8, Dojindo Laboratories, Kumamoto, Japan) at days 0, 1, 3, 5, and 7 in triplicate wells. The optical density at 450 nm were measured using the ELX808 Plate Reader (BioTek).

### Alkaline phosphatase (ALP) and Alizarin red S (ARS) staining and quantification

MSCs were fixed in 95% ethanol. For ALP staining, the BCIP/NBT Staining Kit (CoWin Biotech) was used. ALP activity was measured based on the absorbance at 520 nm using the ALP Assay Kit (Nanjing Jiancheng Bioengineering Institute). For ARS staining, MSCs were incubated with 40 mmol/L filtered 2% Alizarin red buffer (Sigma-Aldrich), followed by 100 nmol/L cetylpyridine for >1 h to quantify calcium-bound ARS by measuring spectrophotometrically the absorbance at 562 nm.

### Oil red O staining and quantification

MSCs were fixed in 10% neutral formalin and washed with 60% isopropanol. Next, Oil red O working solution (Sigma-Aldrich) was added and MSCs were observed using a microscope. Lipid droplets were dissolved in 100% isopropanol for quantitative evaluation, and the absorbance at 500 nm was measured.

### RNA isolation and qRT-PCR

Total RNA was extracted from MSCs using TRIzol (Invitrogen, Carlsbad, CA). RNA was extracted from apoVs and exosomes using the miRNeasy Mini Kit (Qiagen). RNA purity and concentration were measured using a NanoDrop 8000 spectrophotometer (Pierce Thermo Scientific, Waltham, MA). The PrimeScript RT Reagent Kit (#RR037A; Takara, Tokyo, Japan) was used to produce cDNA. For miRNA evaluation, the miDETECT A Track^TM^ qRT-PCR Starter Kit (Ribo Bio) was used to produce cDNA. qRT-PCR was conducted using SYBR Green Master Mix (Yeasen Biotechnology, Shanghai, China) on the ABI Prism 7500 Real-Time PCR Detection System (Applied Biosystems, Foster City, CA). The sequences of the primers are listed in Table S4. Expression levels were normalized to those of GAPDH and U6.

### Western blotting

MSCs were lysed and the protein concentration was determined using the Pierce BCA Protein Assay Kit. Aliquots (40 μg) of protein solutions were resolved by 10% SDS-PAGE (Millipore, Billerica, MA) and transferred to PVDF membranes. The membranes were incubated with diluted anti-RUNX2, anti-PPARγ, anti-GAPDH, anti-β-catenin (ProteinTech), anti-CD9, anti-CD81, anti-Fas, anti-integrin alpha-5, anti-CD44, anti-p65 (Abcam), anti-calreticulin (Cell Signaling Technology), anti-AKT1 (Santa Cruz), anti-p-AKT1, anti-ERK and anti-p-ERK (Abclonal) primary antibodies, followed by the corresponding secondary antibodies (Cell Signaling Technology). Bands were detected using the ECL Kit (NCM bio, Suzhou, China). Images were analyzed using Image J software (National Institutes of Health, Bethesda, MD).

### In vivo implantation of hMSCs

This experiment was approved by the Institutional Animal Care and Use Committee of the Peking University Health Science Center (approval number: LA2021006).

For heterotopic adipose tissue formation, MSCs were cultured for 7 days and seeded on collagen membrane scaffolds (Wuxi Biot Bioengineering Institute, Wuxi, China). For ectopic bone tissue formation, MSCs were combined with β-TCP (RB-SK-005 G). After anesthetization with pentobarbital, the hybrids were imbedded subcutaneously into the backs of nude mice. The implants were harvested after 6 weeks and fixed.

### Mouse model of osteoporosis mouse model and tail-vein injection of apoVs

Eight-week-old female BALB/c mice underwent bilateral OVX to establish a mouse model of estrogen deficiency-induced bone loss. Other eight-week-old female BALB/c mice underwent a sham operation. Eight weeks after surgery, apoV solution (20 μg per 30 g body weight) and PBS (volume equal to that of apoV solution) were administered weekly to the mice via the tail vein. Twelve-month-old female mice also underwent tail-vein injection. The mice were humanely euthanized after 8 weeks and the femurs were dissected and fixed.

### Analysis of apoV distribution in vivo

ApoVs were incubated with 3,3’-dioctadecyloxacarbocyanin perchlorate (DiR, Keygen Biotech, Jiangsu, China) solution and injected into mice via the tail vein. After 24 and 48 h, the livers, lungs, spleens, hearts, kidneys, spines, femurs, mandibular bones, and cranial bones were harvested and scanned using the IVIS Lumina Series III (PerkinElmer, MA).

### Restoration of cranial defects in rat using apoV-PLGA/pDA scaffolds

To produce apoV-PLGA/pDA scaffolds, PLGA/pDA scaffolds were immersed in 1 μg/μL apoV solution for 12 h. The scaffolds were immersed in normal saline, which was replenished daily for assessment of apoVs release by BCA assay. Rats with 5 mm diameter skull defects were subjected to the indicated treatments. After 8 weeks, the cranial bones were harvested and fixed.

### Histological staining and assessment

After fixation for at least 2 days, implants harvested for adipose tissue formation were cut in half. The specimens for H&E, Toluidine Blue and Masson staining were dehydrated and embedded in paraffin, cut into 5 μm sections and stained. Specimens for Oil red O staining were embedded in Tissue-Tek OCT medium, cut into sections 7 mm thick, and stained. Bone tissues were soaked in 0.5 mol/L EDTA for decalcification, sectioned and subjected to H&E staining as described above. Histological sections were observed and images were captured using a microscope.

### Micro-computed tomography (micro-CT)

Images of femurs were obtained at a current of 220 μA, voltage of 60 kV, and exposure time of 1 500 ms. Images of cranial bones were obtained at a current of 500 μA, voltage of 80 kV, and exposure time of 1 500 ms using the Inveon MM system (Siemens, Munich, Germany). Inveon Research Workplace (Siemens) was used to calculate the BV/TV, Tb. Sp, Tb. N, and BMD independently in triplicate. The investigators were blinded to the treatment allocation.

### RNA sequencing analysis

Total RNA (1 μg per sample) was used to construct a miRNA library. After purification of small RNA from total RNA and reverse transcription, the final products (100–120 bp) were sequenced on the BGISEQ-500 platform (BGI-Shenzhen, China). Clean tags were generated using SOAPnuke v. 1.5.2 software (BGI-Shenzhen, China). These tags were mapped to the reference genome and several sRNA databases using Bowtie2^57^. For mapping to the Rfam database v. 13.0, cmsearch^58^ was used. Gene expression levels were quantified by counting the absolute numbers of molecules identified by the unique molecular identifiers^59^. Data processing and enrichment analysis were conducted using R v. 3.1.1 software. Differential expression analysis was performed using the DEGSeq2^60^.

### Statistical analysis

Data are shown as means ± standard deviations. Statistical analysis was performed using Prism software (GraphPad Software, Inc., La Jolla, CA). Between-group comparisons were performed using independent two-tailed Student’s t-tests, and multiple-group comparisons were conducted using one-way ANOVA followed by a Tukey’s *post hoc* tests. A two-tailed value of *P* < 0.05 was considered indicative of statistical significance.

### Supplementary information

Supplementary Information

## Data Availability

The data that support the conclusions of the current study are available from the corresponding authors upon reasonable request.
