# Supplementary material for: Tailored apoptotic vesicles promote bone regeneration by releasing the osteoinductive brake
Source: Int J Oral Sci. 2024 Apr 16;16:31. doi: 10.1038/s41368-024-00293-0 (PMC11021547; doi:10.1038/s41368-024-00293-0)
Supplement: Supplementary file 1 — Supplementary Information [file 41368_2024_293_MOESM1_ESM.pdf]

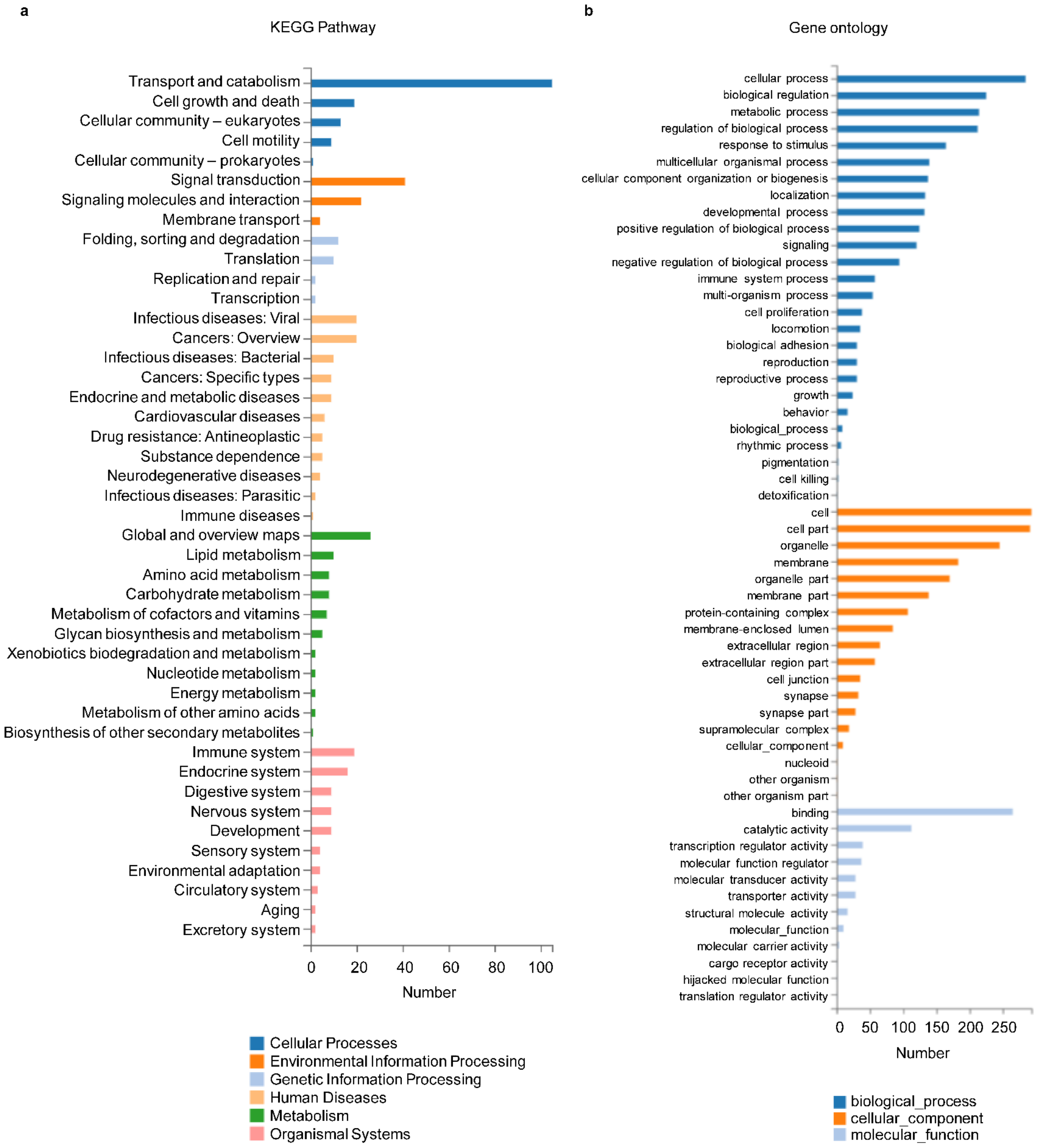

Figure. S1 Enrichment analysis of seven unique miRNAs in MSC-apoVs. (a) KEGG analysis of seven unique miRNAs in MSC-apoVs. The Y-axis represents KEGG terms and the X-axis represents the number of significantly upregulated genes. (b) GO analysis of seven unique miRNAs in MSC-apoVs. The Y-axis represents GO terms and the X-axis represents the number of significantly upregulated genes.

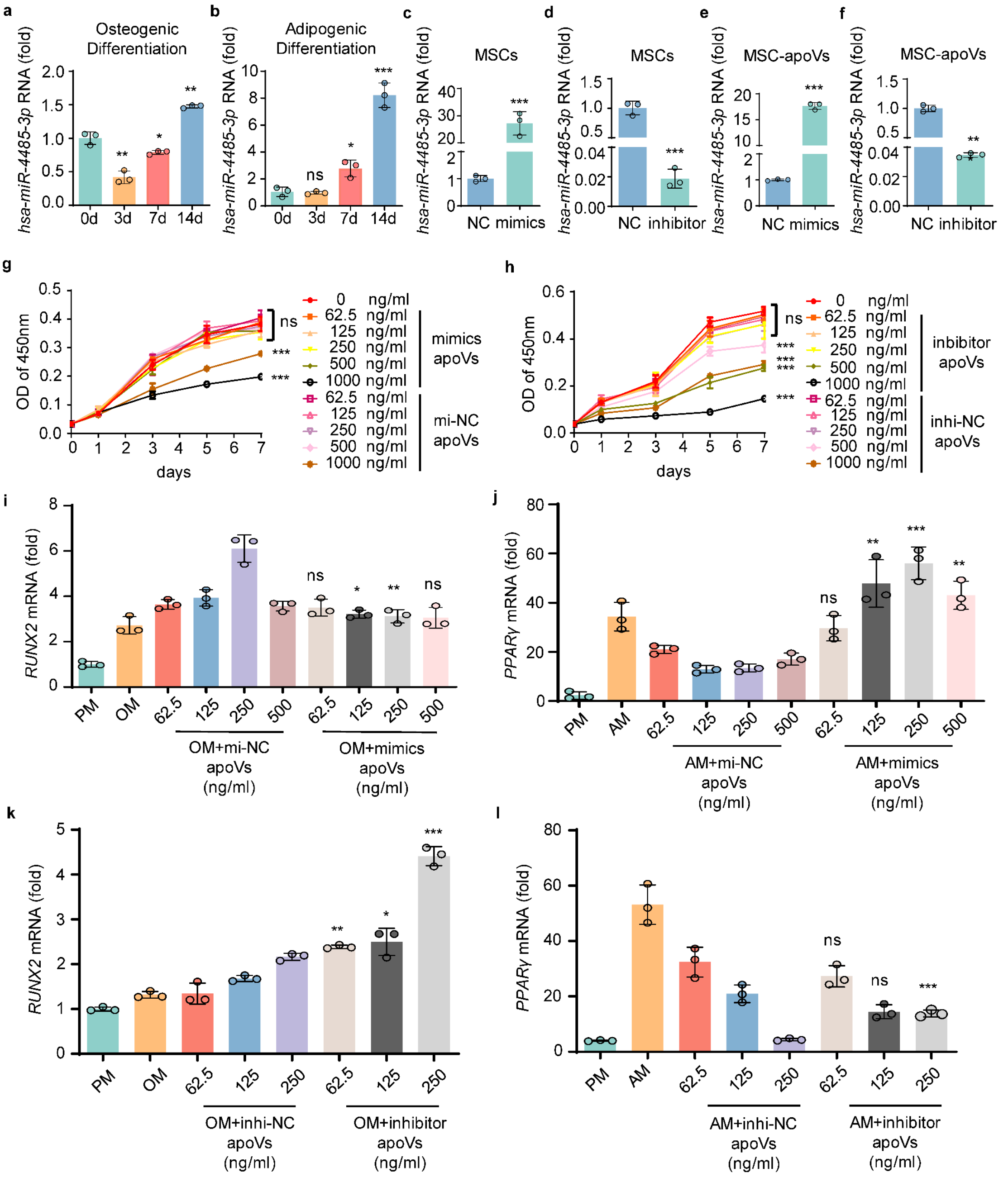

Figure. S2 Screening of apoVs for the most effective concentration in vitro. (a, b) Relative hsa-miR-4485-3p expression level in MSC osteogenic and adipogenic differentiation. The *p*-values are derived from statistical tests comparing each group with group day 0. (c, d, e, f) Relative hsa-miR-4485-3p expression level of transfected MSCs and transfected MSC-apoVs detected by qRT-PCR. (g, h) Cell counting kit-8 assay of MSCs treated with different concentrations of mimics, mi-NC, inhibitor and

inhi-NC apoVs at day 0, 1, 3, 5, 7. The *p*-values are derived from statistical tests comparing each group to its respective control (NC) at the same concentrations. (i, j) The relative *RUNX2* (i) and *PPARγ* expression levels (j) of MSCs treated with different concentrations of mimics and mi-NC apoVs. (k, l) The relative *RUNX2* (k) and *PPARγ* expression levels (l) of MSCs treated with different concentrations of inhibitor and inhi-NC apoVs. The *p*-values are derived from statistical tests comparing each group to its respective control (NC) at the same concentrations. PM, proliferation medium; OM, osteogenic medium; AM, adipogenic medium; mimics apoVs, hsa-miR-4485-3p overexpressed apoVs; mi-NC apoVs, negative control of mimics apoVs; inhibitor apoVs, hsa-miR-4485-3p knockdown apoVs; inhi-NC apoVs, negative control of inhibitor apoVs. Results are presented as the mean ± standard deviation (n=3 per group). ns, $p > 0.05$; *$p < 0.05$; **$p < 0.01$; ***$p < 0.001$.

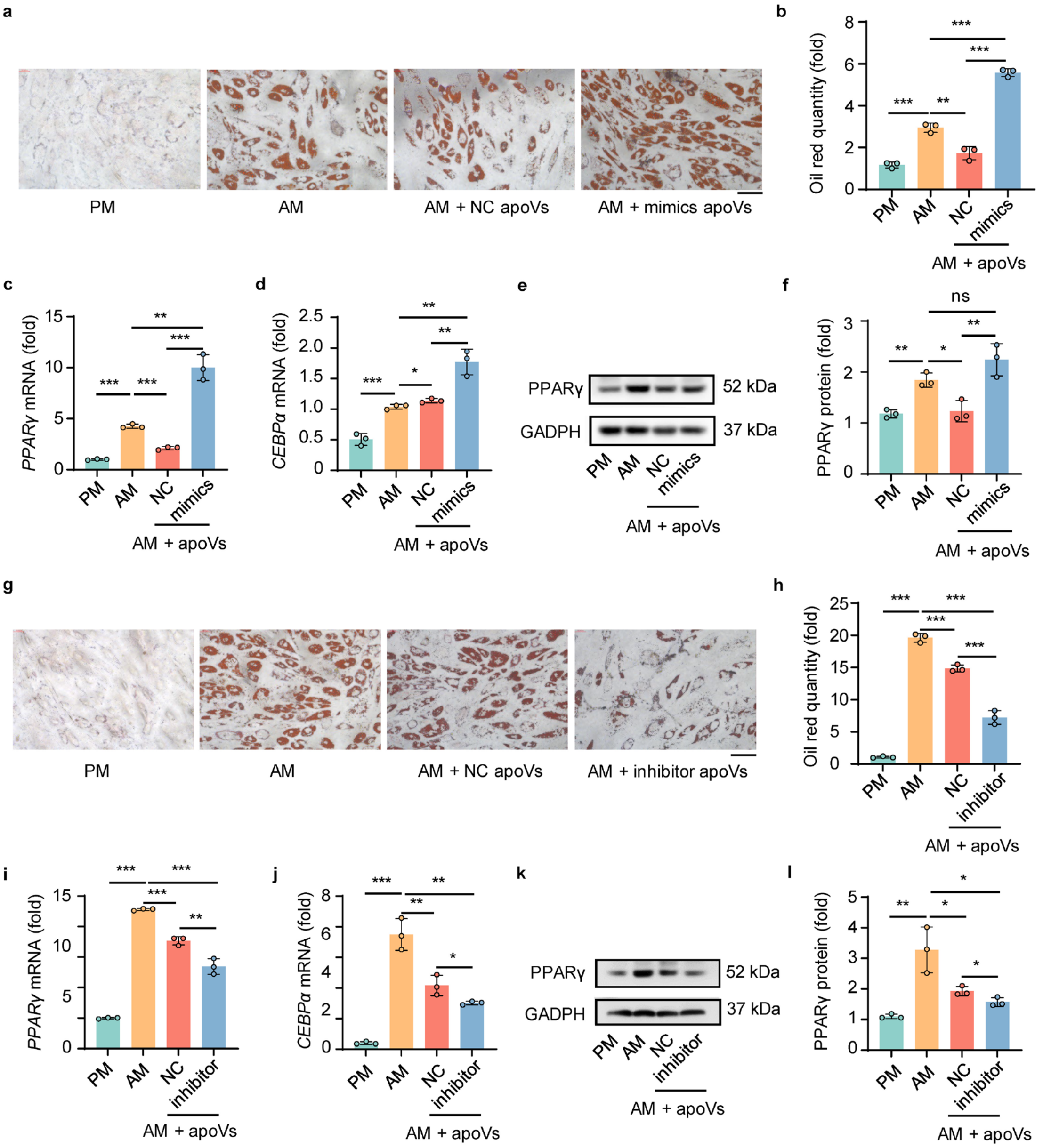

Fig. S3 hsa-miR-4485-3p in apoVs promoted adipogenic differentiation of MSCs *in vitro*. (a, b) Oil red O staining (a) and Oil red O quantity (b) of MSCs treated with PM, AM, AM + NC apoVs and AM + mimics apoVs after adipogenic induction for 14 days. Scale bar, 100 μm. (c, d) The relative mRNA expression levels of the adipogenic markers *PPARγ* (c) and *CEBPα* (d) on day 14 of PM, AM, AM + NC apoVs and AM + mimics apoVs groups determined by qRT-PCR. (e, f) Western blot (e) and protein expression level quantification (f) of PPARγ in PM, AM, AM + NC apoVs, and AM +

mimics apoVs groups after 7 days of treatment. (g, h) Oil red O staining (g) and Oil red O quantity (h) of MSCs treated with PM, AM, AM + NC apoVs and AM + inhibitor apoVs after adipogenic induction for 14 days. Scale bar, 100 μm. (i, j) The relative mRNA expression levels of the adipogenic markers *PPARγ* (i) and *CEBPα* (j) on day 14 of PM, AM, AM + NC apoVs and AM + inhibitor apoVs groups determined by qRT-PCR. (k, l) Western blot (k) and protein expression level quantification (l) of PPARγ in PM, AM, AM + NC apoVs, and AM + inhibitor apoVs groups after 7 days of treatment. PM, proliferation medium; AM, adipogenic medium; NC, negative control apoVs; mimics, hsa-miR-4485-3p overexpressed apoVs. Results are presented as the mean ± standard deviation (n=3). ns, $p > 0.05$; *$p < 0.05$; **$p < 0.01$; ***$p < 0.001$.

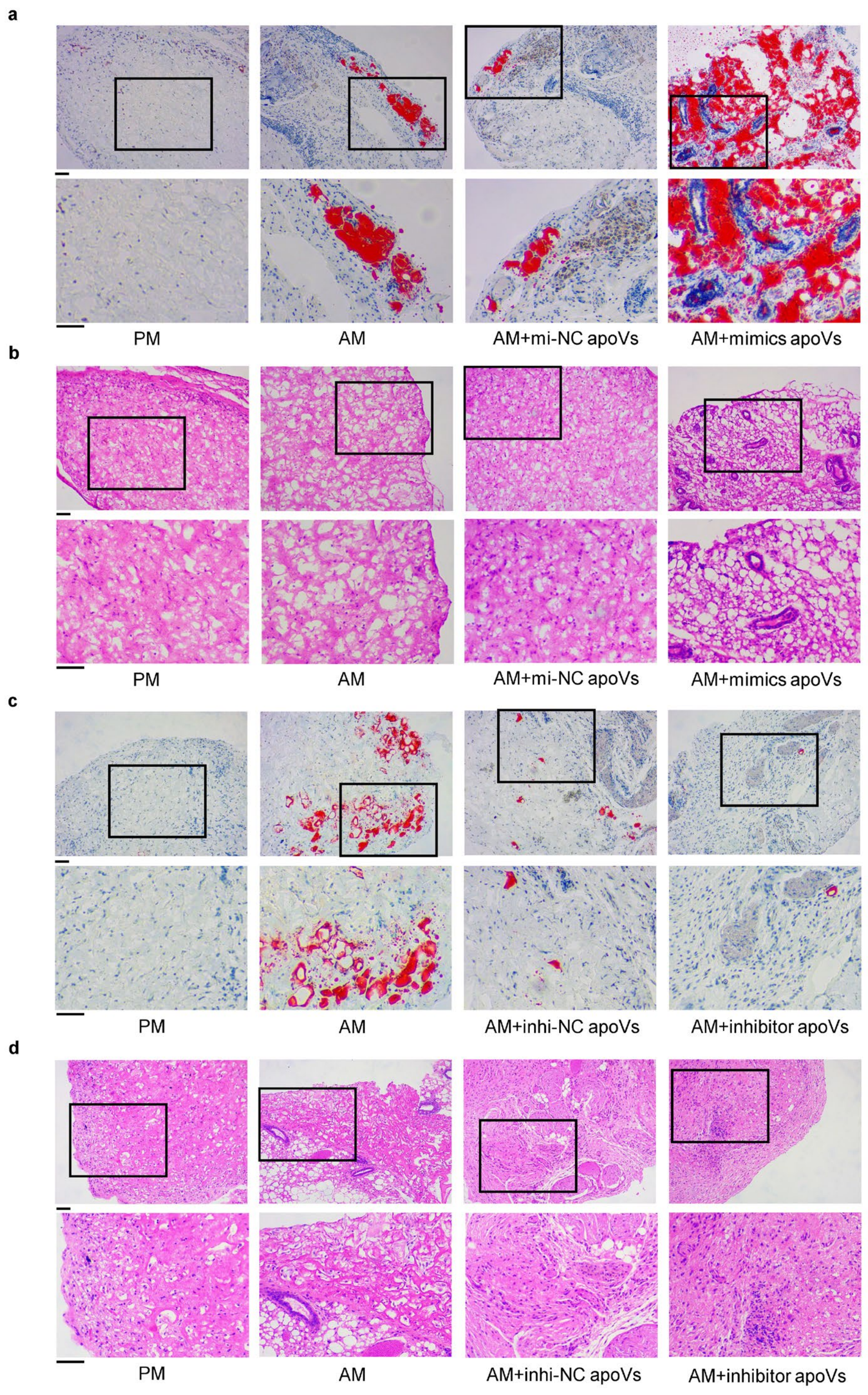

Fig. S4 Histological sections of neo-generated adipose tissue induced by MSCs treated by hsa-miR-4485-3p overexpressed and knockdown apoVs.

Representative microscopic view of Oil Red O staining (a) and H&E staining (b) of PM, AM, AM+mi-NC apoVs, and AM+ mimics apoVs groups; Oil Red O staining (c)

and H&E staining (d) of PM, AM, AM+ inhi-NC apoVs and AM+ inhibitor apoVs groups. The lower panels show the magnified images of the area indicated by the black lines. Scale bar, 100 μm. PM, proliferation medium; AM, adipogenic medium; mimics apoVs, hsa-miR-4485-3p overexpressed apoVs; mi-NC apoVs, negative control of mimics apoVs; inhibitor apoVs, hsa-miR-4485-3p knockdown apoVs; inhi-NC apoVs, negative control of inhibitor apoVs.

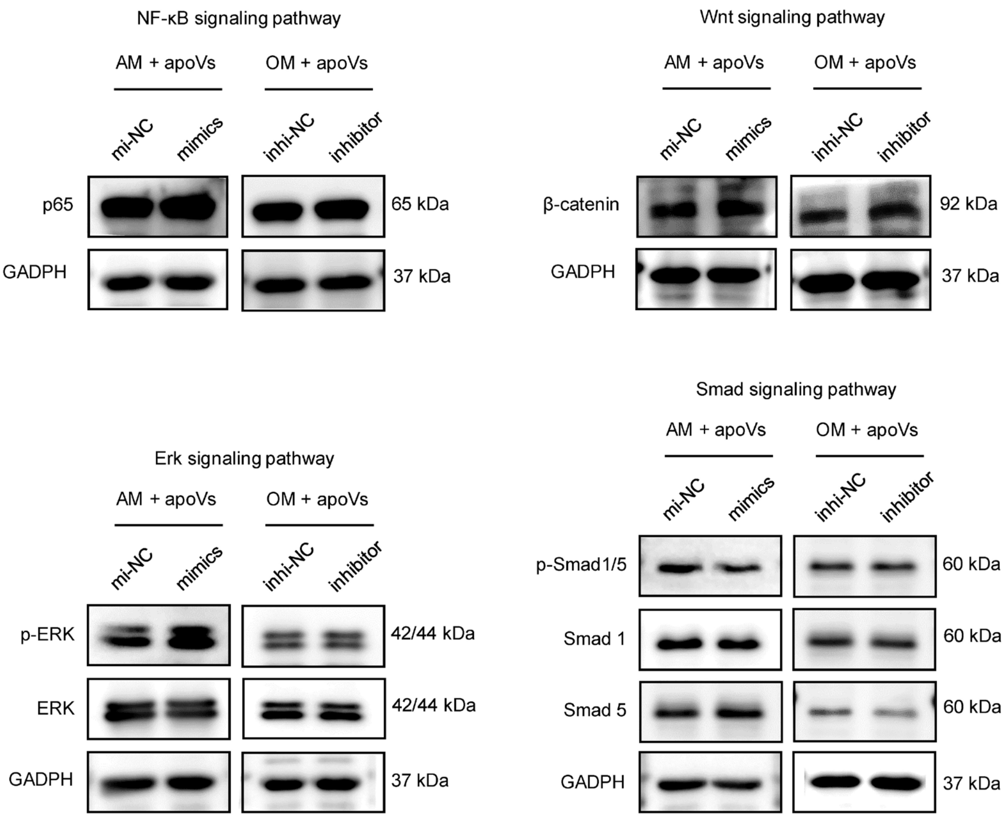

Fig. S5 Western blot of NF-κB, Wnt, ERK and Smad signaling pathways in MSCs treated with mi-NC apoVs, mimics apoVs, inhi-NC apoVs and inhibitor apoVs for 7 days of osteogenic and adipogenic induction. PM, proliferation medium; OM, osteogenic medium; AM, adipogenic medium; mimics apoVs, hsa-miR-4485-3p overexpressed apoVs; mi-NC apoVs, negative control of mimics apoVs; inhibitor apoVs, hsa-miR-4485-3p knockdown apoVs; inhi-NC apoVs, negative control of inhibitor apoVs.

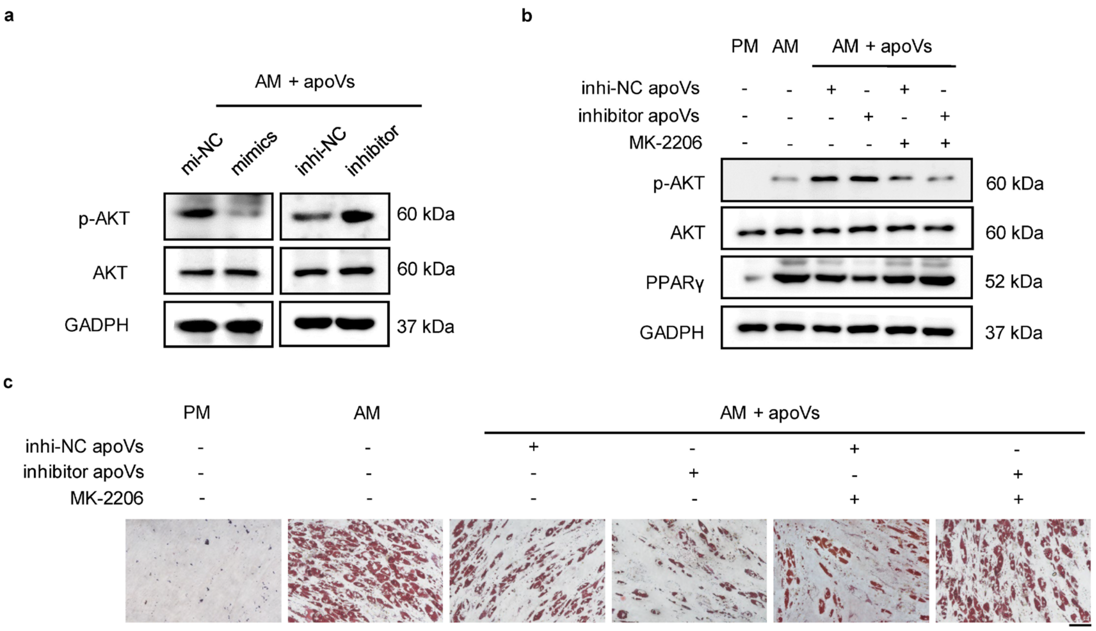

Fig. S6 hsa-miR-4485-3p control MSC adipogenesis through regulating AKT pathway. (a) Western blot showed that hsa-miR-4485-3p in apoVs inhibited the AKT signaling pathway in adipogenesis. (b, c) Western blot (b) and ORS staining (c) results indicated that MK-2206 suppressed the AKT signaling pathway and rescued the effect of hsa-miR-4485-3p in adipogenesis. Scale bar, 100 μm. PM, proliferation medium; AM, adipogenic medium; mimics apoVs, hsa-miR-4485-3p overexpressed apoVs; mi-NC apoVs, negative control of mimics apoVs; inhibitor apoVs, hsa-miR-4485-3p knockdown apoVs; inhi-NC apoVs, negative control of inhibitor apoVs.

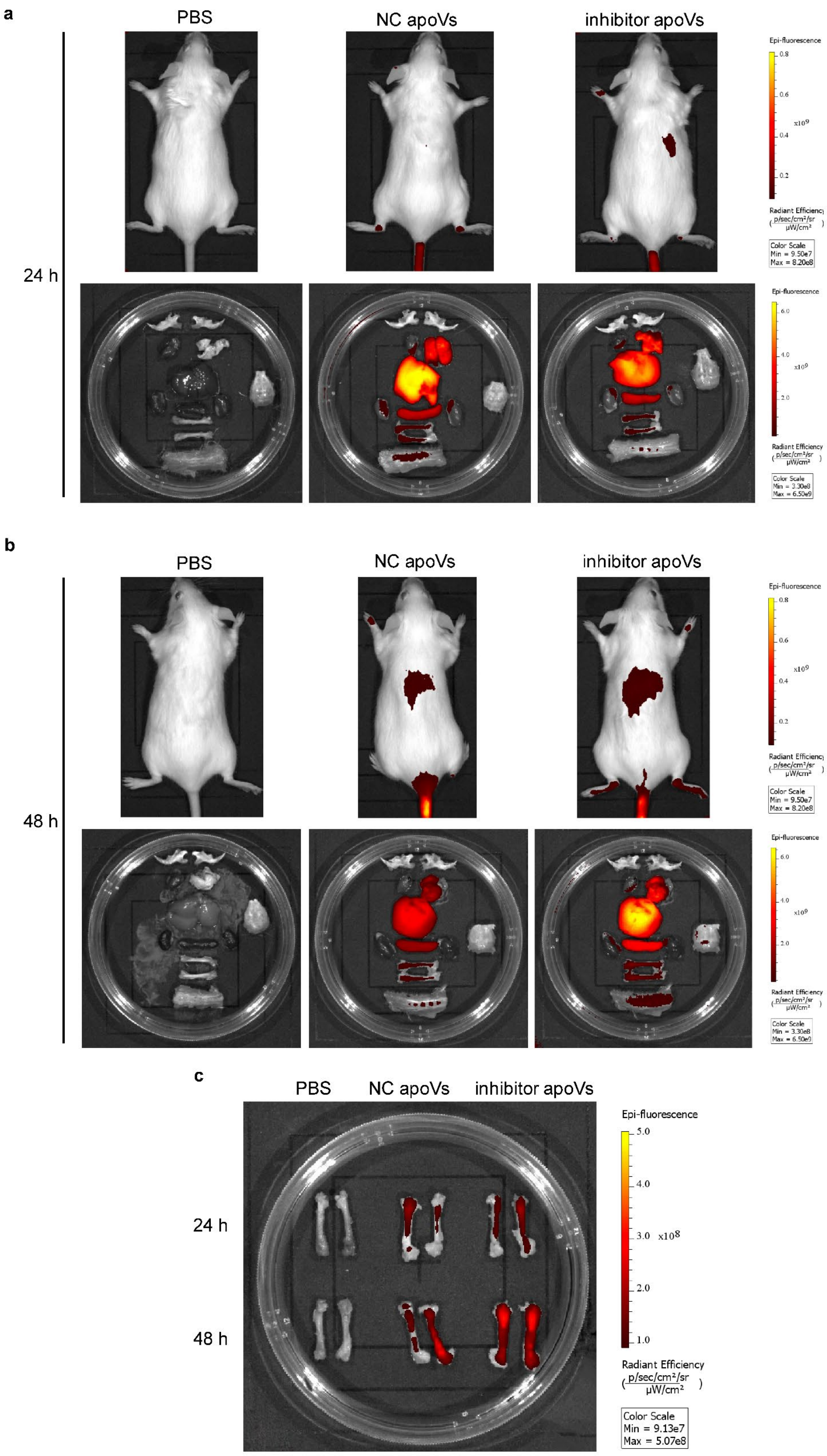

Fig. S7 Biodistribution of apoVs after tail vein injection. The fluorescent signal of major organs and whole-body fluorescence imaging at 24h (a) and 48h (b) after tail vein injection. (c) The florescent signal of femurs in PBS, NC apoVs and inhibitor

apoVs groups at 24h and 48h. inhibitor apoVs, hsa-miR-4485-3p knockdown apoVs; NC apoVs, negative control of inhibitor apoVs.

Table S1 The 20 most abundant miRNAs identified in hBMMSC-apoVs and their expression level

Gene ID	Cells	ApoVs	Exosomes
hsa-miR-22-3p	132491.667	105797.667	111595.667
hsa-miR-100-5p	112333.667	102847	74420
hsa-miR-21-5p	95380.667	98008.667	90791.333
hsa-let-7i-5p	124504.333	98007.333	37495.333
hsa-let-7b-5p	123377.333	91935	23442.667
hsa-let-7a-5p	126360.333	91153.667	35223
hsa-miR-143-3p	105951.333	86088.333	95632
hsa-miR-34a-5p	96550.667	85775.667	63177.667
hsa-miR-222-3p	106939.333	82671.667	28709.333
hsa-miR-26a-5p	91675	79215.667	16790.333
hsa-miR-181a-5p	79972	76709.333	22938.333
hsa-miR-27a-3p	87937	70384.333	82235
hsa-miR-23a-3p	63521	56992	71993.667
hsa-miR-127-3p	46145.333	50786.333	16357.333
hsa-let-7f-5p	77234.333	48267.667	19329
hsa-miR-199a-5p	67910.333	43716.667	10702
hsa-miR-221-3p	28930.333	43577.667	49590.667
hsa-miR-125b-5p	25631	43447.333	5313.667
hsa-miR-99a-5p	39714.333	41139.333	14473
hsa-miR-24-3p	55015.667	39233.333	66710.667

Table S2 The 20 most abundant miRNAs identified in hASC-ApoVs and their expression level

Gene ID	Cells	ApoVs	Exosomes
hsa-miR-22-3p	103849.333	119536	183016
hsa-miR-100-5p	92907.667	112760.667	102477.667
hsa-miR-21-5p	102538.667	106514	129707
hsa-let-7i-5p	88403.333	98985	111114.333
hsa-miR-181a-5p	61844.667	92285.667	85320.333
hsa-miR-143-3p	77482.333	89452.333	109847
hsa-let-7a-5p	82212	88077.667	101884
hsa-miR-222-3p	93963.667	87005.333	86189
hsa-miR-26a-5p	82405.333	84365.667	95245.667
hsa-let-7b-5p	67976.667	69047.333	91950.333
hsa-miR-27a-3p	46641.667	59996.667	83012
hsa-miR-23a-3p	40583.333	57079.333	66693.333
hsa-miR-10a-5p	69235	56374	16118
hsa-miR-34a-5p	53649.333	54820	74154.333
hsa-let-7f-5p	54107.667	54641.333	74665.333
hsa-miR-99a-5p	51195.667	47051.667	52707.333
hsa-miR-127-3p	47376.667	44649.667	52586
hsa-miR-31-5p	43305.333	44024.667	31271.667
hsa-miR-24-3p	52467	41673.667	54037.333
hsa-miR-92a-3p	48042.333	40832.667	31484

Table S3. The quantity of DEGs identified during Small RNA sequencing analysis.

Compared Groups	Down	Up	Total
hB-apoVs vs. hBMMSCs	66	71	137
hB-apoVs vs. hB-exos	123	240	363
hA-apoVs vs. hASCs	67	53	120
hA-apoVs vs. hA-exos	67	71	138

Adjusted p-value < 0.05 in combination with fold change≥2 was set to identify the differential expression.

Table S4. Sequences of PCR primers.

Name	Forward Primer (5′–3′)	Reverse Primer (5′–3′)
hsa-miR-4485-3p	TGGATTTAACGGCCGCG	TATGGTTCTTGACGACTGGTTGAC
U6	CGCTTCGGCAGCACATATAC	TTCACGAATTTGCGTGTCATC
OCN	AGCCACCGAGACACCATGAGA	GGCTGCACCTTTGCTGGACT
ALP	GACCTCCTCGGAAGACACTC	TGAAGGGCTTCTTGTCTGTG
RUNX2	ATGGGATGGGTGTCTCCACA	CCACGAAGGGGAACTTGTC
BMP2	CCTTGCGCCAGGTCCTTTGA	GGTCGACCTTTAGGAGACCGCA
C/EBPα	CGCAAGAGCCGAGATAAAGC	CACGGCTCAGCTGTTCCA
PPARγ	CGAGACCAACAGCTTCTCCTTC	TTTCAGAAATGCCTTGCAGTGG
GAPDH	CGGACCAATACGACCAAATCCG	AGCCACATCGCTCAGACACC
